# The therapeutic promises of Lianhuaqingke in the mice model of coronavirus pneumonia (HCoV-229E and SARS-CoV-2)

**DOI:** 10.1186/s13020-021-00513-3

**Published:** 2021-10-18

**Authors:** Mingye Wang, Wenyan Li, Wenwen Cui, Yuanyuan Hao, Yao Mi, Hongtao Wang, Yunlong Hou, Zhenhua Jia

**Affiliations:** 1 College of Integrated Traditional Chinese and Western Medicine, Hebei Medical University of Chinese Medicine, No.326, The South of Xinshi Street, Shijiazhuang, 050091 Hebei China; 2 Shijiazhuang Yiling Pharmaceutical Co., Ltd, No.238, The South of Tianshan Street, Shijiazhuang, 050035 Hebei China; 3 National Key Laboratory of Collateral Disease Research and Innovative Chinese Medicine, No.238, The South of Tianshan Street, Shijiazhuang, 050035 Hebei China; 4 Shijiazhuang Compound Traditional Chinese Medicine Technology Innovation Center, No.238, The South of Tianshan Street, Shijiazhuang, 050035 Hebei China; 5 Hebei Yiling Hospital, Shijiazhuang, 050035 Hebei China

**Keywords:** Lianhuaqingke (LHQK), Human coronaviruses (HCoVs), Immunomodulation, Anti-HCoVs therapy

## Abstract

**Background:**

Lianhuaqingke (LHQK) has been approved for the treatment of acute tracheobronchitis and exerts a broad-spectrum antiviral effect in our previous study.

**Methods:**

Acute pneumonia caused by HCoV-229E was modeled in BALB/c mice. The anti-viral effect of LHQK was assessed by measuring the lung index and virus titer of lung tissues. The expression levels of pro-inflammatory cytokines in lung tissues and peripheral blood were measured by ELISA. The morphological changes of lung tissues were observed by H&E staining. The subsets of Th cells were assayed by the flow cytometry, including Th0, Th1, Th2, Treg, and Th17. The expression level of MUC5AC in 16HBE cells treated with TNFα was measured by ELISA. Immunofluorescence staining for β-IV tubulin was used to identify the airway epithelial ciliary in the condition-cultured RTE cells treated with TNFα. The direct antiviral effect of LHQK was assessed in vitro in Vero E6 infected by SARS-CoV-2, validated in vivo in the COVID-19 model of hACE2 transgenic mouse by detecting the lung index, the SARS-CoV-2 virus load, and the morphological changes of lung tissues.

**Results:**

LHQK reduced the weight loss and the lung index by inhibiting the HCoV-229E replication and reducing the expression of pro-inflammatory cytokines in lung tissues. An assay for the Th cell subsets in peripheral blood revealed that LHQK could reduce the ratio of Th1/Th2 and increase the Treg/Th17 ratio in a dose-dependent way, which indicated that LHQK could coordinate the Th-mediated immune responses against the virus. In in vitro injury by TNFα, LHQK inhibited MUC5AC expression in 16HBE cells and increased the number of β-IV tubulin positive staining cells in the condition-cultured RTE cells. In the SARS-CoV-2-infected mice, LHQK could reduce weight loss, inhibit viral replication, and alleviate lung tissue damage.

**Conclusions:**

Our results demonstrate that LHQK exerts therapeutic effects on pneumonia caused by HCoVs (HCoV-229E and SARS-CoV-2) in mice, and that the anti-HCoV effects might depend on its immunomodulatory capacities. All these results suggest that LHQK serves as a potential adjuvant for anti-HCoV therapies.

## Background

Human coronaviruses (HCoVs) are a group of enveloped viruses with positive single-stranded RNA genomes, which are named for their coronary appearance [1]. Until now, 7 known strains of coronaviruses, including the novel SARS-CoV-2 coronavirus, are infectious to humans to cause respiratory diseases with mild to severe outcomes [2]. Depending on the severity of the viral infection, HCoVs are technically divided into two categories, mildly and highly pathogenic HCoVs. The mildly pathogenic HCoVs are known to cause mild upper respiratory symptoms, presumably contributing to 15–30% common cold in humans [3], and severe respiratory infections rarely occur in infants, elderly people, or immunocompromised patients [4]. On the contrary, the highly pathogenic HCoVs tend to lead to fatal respiratory failure and acute respiratory distress syndrome. Over the past two decades, we have witnessed outbreaks of three zoonotic and highly pathogenic HCoVs. However, the approaches for prevention and treatment of HCoVs infection are limited for short of vaccines or specific antiviral drugs [5, 6]. It is becoming increasingly urgent to find a practical treatment for those known HCoVs or even for future reemergence or the novel emerging virus [7].

The novel SARS-CoV-2 is spreading around the world, averaging about 550,000 new cases and almost 9000 deaths per day. Recently, the spread of SARS-CoV-2 in China has been effectively controlled. On the one hand, rigid segregation and social distance limit the virus spread. On the other hand, Traditional Chinese medicines have received broad adoption in the absence of effective antiviral agents, especially at the beginning of breakout, and contribute to the alleviation of the clinical symptoms and the prevention of the condition’s progression [8–10]. Lianhuaqingke (LHQK) is a novel Chinese patent medicine produced by Shijiazhuang Yiling Pharmaceutical Co., Ltd and is launched on market during the pandemic of COVID-19, which has been prescribed to treat acute tracheobronchitis especially by relieving the symptoms of cough and expectoration. Although the HCoVs pathogenicity is dramatically different, the severe cases generally manifest lower respiratory infection accompanied with hyperthermia, cough, and expectoration. In addition to antiviral agents, the treatment guideline for severe cases will suggest taking some symptomatic treatment drugs to reduce serious complications [11].

In the present study, we evaluated the therapeutic effects of LHQK on acute coronavirus pneumonia caused by HCoV-229E and SARS-CoV-2 in mice. The results proved that LHQK exerted anti-viral and anti-inflammatory effects to treat coronavirus pneumonia, making it promising as a novel strategy for controlling coronavirus infection in clinical practice.

## Materials and methods

### Reagent preparation

The Lianhua-Qingke tablets material (LHQK, Lot No. A1401001) and The Lianhua-Qingwen capsule material (LHQW, Lot No. B2001013) were provided by Shijiazhuang Yiling Pharmaceutical Co., Ltd. (Shijiazhuang, China). The tables of LHQK were dissolved in pure water to 143 mg/mL, 286 mg/mL, and 572 mg/mL before use. The capsules of LHQW were dissolved in pure water to 156 mg/mL before use.

**Fig. 1:**
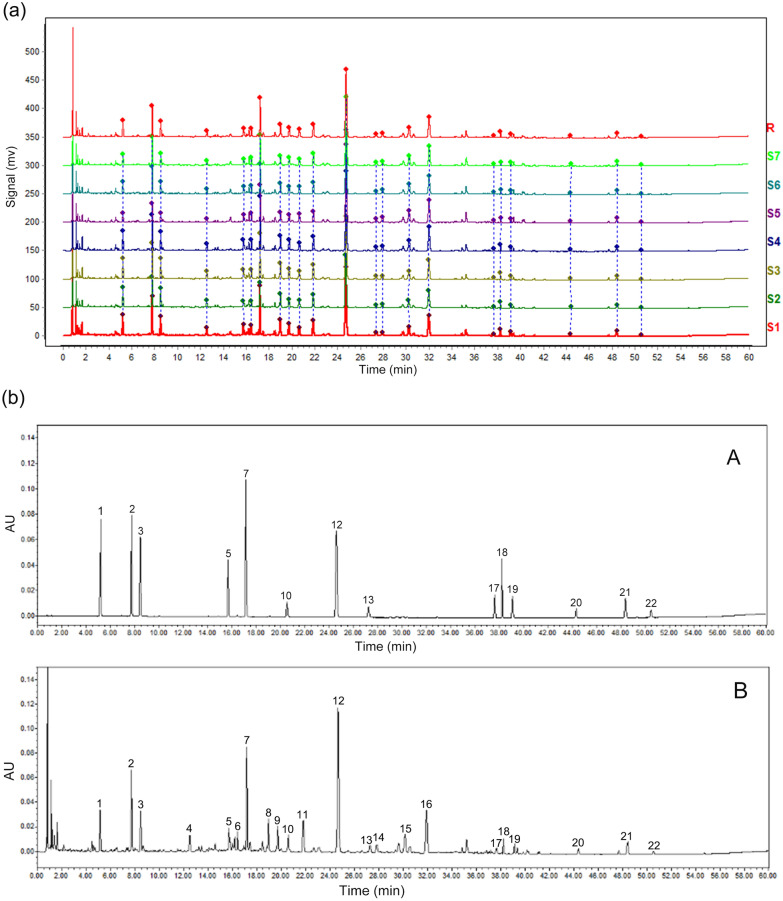
**a** Chromatograms of seven batches of Lianhua Qingke. **b** The typical chromatogram of mixed standards (**A**) and Lianhua Qingke (**B**)

The control sample (R) and seven batches of Lianhua-Qingke tablets samples (S1: A1912001, S2: A1911001, S3: A1911002, S4: A1911003, S5: A1812001, S6: A1812002, S7: A1812003) were analyzed as the standard characteristic fingerprint of Lianhua-Qingke tablets (Fig. 1a). 22 common characteristic peaks were applied to evaluate the similarity of 7 batches of Lianhua-Qingke tablets. The chromatograms were used to generate the similarity values between 0.969 to 1.0 (Table 1), indicating a good consistency among samples in similarity evaluation. The typical chromatogram of Lianhua-Qingke tablets and mixed standards were shown in Fig. 1b. 14 out of 22 peaks were identified by comparing the retention time and UV spectrum of each peak with those of standard compounds: Neochlorogenic acid (1), chlorogenic acid (2), cryptochlorogenic acid (3), isoforsythiaside (5), phillygenin (7), hesperidin (10), baicalin (12) as the reference, arctiin (13), aloe-emodin (17), glycyrrhizic acid ammonium salt (18), rhein (19), emodin (20), 1,8-dihydroxy-3-methylanthraquinone (21), physcion (22). The mixed standard solutions were used to determine the concentration of 14 components in Lianhua-Qingke tablets (Table 2).

**Table 1:** Similarity values of the control sample and seven batches of LHQK samples

No.	S1	S2	S3	S4	S5	S6	S7	R
S1	1.000	0.992	0.999	0.997	0.976	0.991	0.987	0.996
S2	0.992	1.000	0.994	0.988	0.969	0.989	0.985	0.992
S3	0.999	0.994	1.000	0.995	0.972	0.989	0.985	0.995
S4	0.997	0.988	0.995	1.000	0.988	0.995	0.994	0.999
S5	0.976	0.969	0.972	0.988	1.000	0.994	0.995	0.990
S6	0.991	0.989	0.989	0.995	0.994	1.000	0.999	0.998
S7	0.987	0.985	0.985	0.994	0.995	0.999	1.000	0.997
R	0.996	0.992	0.995	0.999	0.990	0.998	0.997	1.000

**Table 2:** Concentration of 13 components in seven batches of LHQK

Peak number	Content (mg/g)						
	1912001	1911001	1911002	1911003	1812001	1812002	1812003
1	2.040	2.062	2.148	1.957	0.885	1.229	1.140
2	3.431	2.644	3.146	3.108	1.672	2.429	2.521
3	2.005	1.998	2.087	1.926	0.896	1.231	1.176
5	2.294	1.545	2.034	2.277	1.592	1.518	1.364
7	7.175	3.525	6.560	7.869	5.443	4.334	4.421
10	10.226	9.572	10.499	9.935	10.414	9.103	8.236
12	10.430	7.962	9.502	12.055	11.680	9.514	10.268
13	6.521	5.164	5.499	6.871	6.591	4.816	6.115
17	0.212	0.142	0.162	0.194	0.194	0.167	0.164
18	0.617	0.538	0.638	0.593	0.444	0.428	0.347
19	0.470	0.305	0.343	0.413	0.418	0.365	0.366
20	0.283	0.169	0.205	0.254	0.252	0.217	0.221
21	0.546	0.350	0.394	0.477	0.481	0.425	0.430
22	0.138	0.078	0.095	0.116	0.115	0.105	0.104

### Cell lines and virus

The African green monkey kidney epithelial (Vero E6) cells, human lung fibroblasts (MRC-5) cells and human bronchial epithelial (16HBE) cells were cultured in Dulbecco’s Modified Eagle’s medium (DMEM; Hyclone, Shanghai, China) supplemented with 10% fetal bovine serum (FBS; Sciencell, USA) and 1% penicillin/streptomycin at 37 ℃ with 5% CO_2_. HCoV-229E (Lot No. ATCC-VR-740) was presented by Hunan Center for Disease Control and Prevention. SARS-CoV-2 was provided by the biosafety level 3 (BSL-3) Laboratory of Guangzhou Customs Technical Center (State Key Lab of Respiratory Disease, Guangzhou, China). HCoV-229E was propagated in MRC-5 cells and SARS-CoV-2 was propagated in the Vero E6 cells, and viral titer was determined by 50% tissue culture infective dose (TCID_50_) according to the cytopathic effect (CPE) by Reed–Muench method.

### Cytotoxicity assay

The cytotoxic effects of the LHQK on the viability of Vero E6 were determined by the methyl thiazolyl tetrazolium (MTT) assay. 2 × 10^5^ Vero E6 cells per well were cultured in 96-well plates overnight, treated with different concentrations of LHQK for 72 h. Cells were stained with MTT solution (Promega, Shanghai, China; G3580) at 37 ℃ for 3 h. The absorbance was recorded at 490 nm using Multiskan Spectrum reader (Thermo Fisher, USA).

### Cytopathic effect (CPE) inhibition assay

The Vero E6 cell monolayers were plated in 96-well plates overnight and then inoculated with 100 TCID_50_ of SARS-CoV-2 at 37 ℃ for 2 h, and then removed the medium and added different concentrations of LHQK for 72 h, observing the CPE of the infected cells under the microscope. The median inhibitory concentration (IC_50_) of LHQK was calculated using the Reed–Muench method.

### Cell viability and MUC5AC determination in vitro

According to the previous study, TNF-α (Peprotech, Inc., Rocky Hill, NJ) was dissolved directly in DMEM at a concentration of 125 ng/mL [12]. The 16-HBE cells were cultured in serum-free DMEM for 12 h to maintain a low basal level of MUC5AC expression and then treated with TNF-α in the absence or presence of LHQK (0.25–250 μg/mL) for 24 h. Cell viability was measured by MTT assay. The cell culture supernatant of 16-HBE cells was collected for ELISA analysis of MUC5AC according to the manufacturer’s recommendations.

### Establishment of HCoV-229E-infected mouse model and treatment

Total 120 BALB/c mice (half male and half female, 10–14 g) were obtained from Hunan SJA Laboratory Animal Co., Ltd. (Hunan, China. Production Certificate No. SCXK (Xiang) 2019-0004). Mice were divided into six groups (n = 20 per group) by sex and weight, consisting of normal control group (normal), model control group (model), LHQW group (LHQW), 2.86 g/kg LHQK group (LHQK Low), 5.72 g/kg LHQK group (LHQK Medium), and 11.44 g/kg LHQK group (LHQK High). The mice were maintained under standard laboratory conditions contained a 12-h light/dark cycle with free access to food and water. The experimental procedures and animal welfare were conducted with approval from the Ethics Review Committee for Animal Experimentation of Drug Safety Evaluation and Research Center of Hunan Province.

The HA titer of HCoV-229E was 6log2, and the mice from groups of the model, LHQW, LHQK Low, LHQK Medium, and LHQK High were intranasally instilled with 60 μL HCoV-229E for 2 consecutive days. Animals of the normal control group were intranasally instilled with 60 μL medium for 2 consecutive days. Following the HCoV-229E infection, the mice from groups of LHQW, LHQK Low, LHQK Medium, and LHQK High received medicine at the volume according to bodyweight (20 mL/kg) once daily for 7 consecutive days. The mice of the normal control group and the model control group were orally administrated with distilled water.

### Determination of bodyweight and lung index in lung tissues

The bodyweight of HCoV-229E-infected mice was recorded every day. After the last administration of drugs, all mice were sacrificed by cervical dislocation. The whole lung tissues of HCoV-229E-infected mice were harvested and weighed. The lung index was calculated via wet lung mass (Lung index = Lung mass/Body mass × 100%) [13].

### Hemagglutination (HA) titer determination of HCoV-229E in vivo

The whole lung tissues of HCoV-229E-infected mice were harvested and dried on filter paper. The homogenate of whole lung tissue was prepared according to the ratio of lung mass (g) to 0.9% sodium chloride injection (ML) at 1:9. Blood was collected from healthy chickens by vein and diluted to 1% the suspension of chicken red blood with physiological saline and stored at 4 ℃. 50 µL of physiological saline was added into 12 wells (labeled from 1 to 12) of 96 well V-shaped coagulation plate. As an example, 50 µL the lung tissue homogenate was added into well A1 and mixed gently, following with two-fold serial dilutions to A11. The well A12 served as control. 50 µL 1% the suspension of chicken red blood was added to each well, mixed on a plate shaker for 1 min. Finally, the plate was kept at room temperature for 15–20 min. The HA titer was calculated as the highest dilution of homogenates showing hemagglutination.

### Histopathological analysis

HCoV-229E-infected mice were sacrificed at 48 h and D8 after medication and the lungs were harvested. The lung tissue specimens were fixed in 10% formaldehyde, embedded in paraffin, and cut into 3–5 µm thickness. The morphological and histopathological changes of lung tissue were evaluated by Hematoxylin & eosin (H&E) staining.

### ELISA assays in lung tissues and peripheral blood of HCoV-229E-infected mice

HCoV-229E-infected mice were sacrificed at 48 h and D8 after medication and the lungs were harvested. According to the manufacturer’s recommendations, we used commercial ELISA kits (MEIMIAN, Jiangsu) to determine the level of inflammatory cytokines of lung tissues and TCIRG1, IRF of peripheral blood.

### Classification of peripheral blood T helper cells of HCoV-229E-infected mice by flow cytometry

100 µL of EDTA-treated peripheral blood was poured into standard polystyrene flow cytometry tubes. The antibody master mix that was prepared by CD25 (Lot No. 2018381), Foxp3 (Lot No. 2105941), CD4 (Lot No. 2115770), and IL-17A (Lot No. 1995433) antibodies according to manufacturer’s instructions was added and mixed lightly by vortexing, then incubated for 30 min at room temperature (RT) in the dark. 2 mL of 1× RBC Lysis Buffer was added and mixed with a lesser extend of vortexing, then incubated for 10 min. Afterwards, the cells were centrifuged at 500×*g* for 5 min at RT, washed twice in 3 mL phosphate buffered saline (PBS). Cells were resuspended with Cell Staining Buffer and adjusted the cell density. The samples were finally analyzed by flow cytometry.

### Immunofluorescence assay in vitro

Normal rat bronchial primary epithelial cells (RTE) were isolated from the bronchus of rats, and seed into transwells (Corning, USA) at a density of 2 × 10^5^ cells in 500 μL BEBM (Lonza, Switzerland) per transwell. The RTE cells were cultured for about 5 days with BEGMTM Bronchial Epithelial Cell Growth Medium BulletKitTM (Lonza, Switzerland), and then the medium was removed when the transepithelial electrical resistance of cells reached 1000 Ω/cm^2^. After washing the cells twice gently with PBS, the RTE cells were cultured for about 5 days with B-ALI™ Bronchial Air–Liquid Interface Medium BulletKit™ (Lonza, Switzerland). RTE cells were then treated with TNF-α (125 ng/mL) in the absence or presence of LHQK (0.025–250 μg/mL) for 24 h. Immunofluorescence staining for β-IV tubulin was performed according to the manufacturer’s instructions.

### Establishment of a SARS-CoV-2-infected hACE2 transgenic mouse model and treatment

To investigate the curative effect of LHQK, we established a SARS-CoV-2-infected hACE2 transgenic mice model. Twelve hACE2 transgenic mice were intranasally inoculated with 10^5^ Median Tissue Culture Infectious Dose (TCID_50_) of SARS-CoV-2 (50 μL) on day 0, and were divided into two groups by weight: the model group and the LHQK group. The SARS-CoV-2-infected hACE2 transgenic mice of the LHQK group received medicine at the volume according to bodyweight (1 mL/100 g) once daily for 5 consecutive days beginning on the day of inoculation (day 0), and the model group was orally administrated with distilled water. The dose of LHQK was 14.67 g/kg. The bodyweight of SARS-CoV-2-infected hACE2 transgenic mice was recorded every day. All mice were euthanized on day 5, and the whole lung tissues of SARS-CoV-2-infected hACE2 transgenic mice were collected for histopathological analysis and determination of viral load. For histology, the lung tissue specimens were fixed in 10% formaldehyde, embedded in paraffin, and cut into 3–5 µm thickness. Sections of the fixed tissue (3–5 µm thickness) were stained with Hematoxylin & eosin (H&E) staining.

### Viral load determination of SARS-CoV-2 in vivo

Viral load detection was performed by RT-qPCR. The total RNA of the lungs was extracted from lung homogenate by using the Eastep^®^ Super Total RNA Extraction Kit (Promega, Shanghai, China). The homogenate of whole lung tissue was prepared by using an electric homogenizer. The reverse transcription was performed using the GoScript™ Reverse Transcription System (Promega, Shanghai, China) according to the manufacturer’s instructions. RT-qPCR reactions were performed in duplicates using the GoTaq^®^ qPCR Master Mix (Promega, Shanghai, China) according to the manufacturer’s protocol. The primer sequences used for qRT-PCR were targeted against the envelope (E) gene of SARS-CoV-2 and were as follows: forward: 5′-TCGTTTCGGAAGAGACAGGT-3′; reverse: 5′-GCGCAGTAAGGATGGCTAGT-3′. The amplification condition was set at 40 cycles at 95 ℃ for 15 s and 60 ℃ for 30 s using a LightCycler^®^ 96 Instrument (Roche Life Science, Penzberg, Germany). Viral load results for each mouse’s lung were expressed as log_10_-transformed numbers of genome equivalent copies per ml by comparing the C_t_ values to the standard curves.

### Statistical analysis

The results were presented as mean ± SD. Statistical analysis was performed using GraphPad Prism 7.0 software and SPSS 21.0 statistical software. The data of normality and variance homogeneity were assessed by Leven’s test. If there was no statistical significance (P > 0.05), one-way analysis of variance (ANOVA) was assessed for statistical analysis. If the ANOVA was statistically significant (P ≤ 0.05), the LSD test (parameter method) was used for comparative analysis. If the variance was uneven (P ≤ 0.05), the Kruskal–Wallis test was used for statistical analysis. If the Kruskal–Wallis test was statistically significant (P ≤ 0.05), the Dunnett’s Test (nonparametric method) was used for comparative analysis. ^+^P ≤ 0.05, ^++^P ≤ 0.01 vs normal group. *P ≤ 0.05, **P ≤ 0.01 vs model group.

## Results

### Cytotoxic and antiviral effect of LHQK against SARS-CoV-2 in vitro

**Fig. 2:**
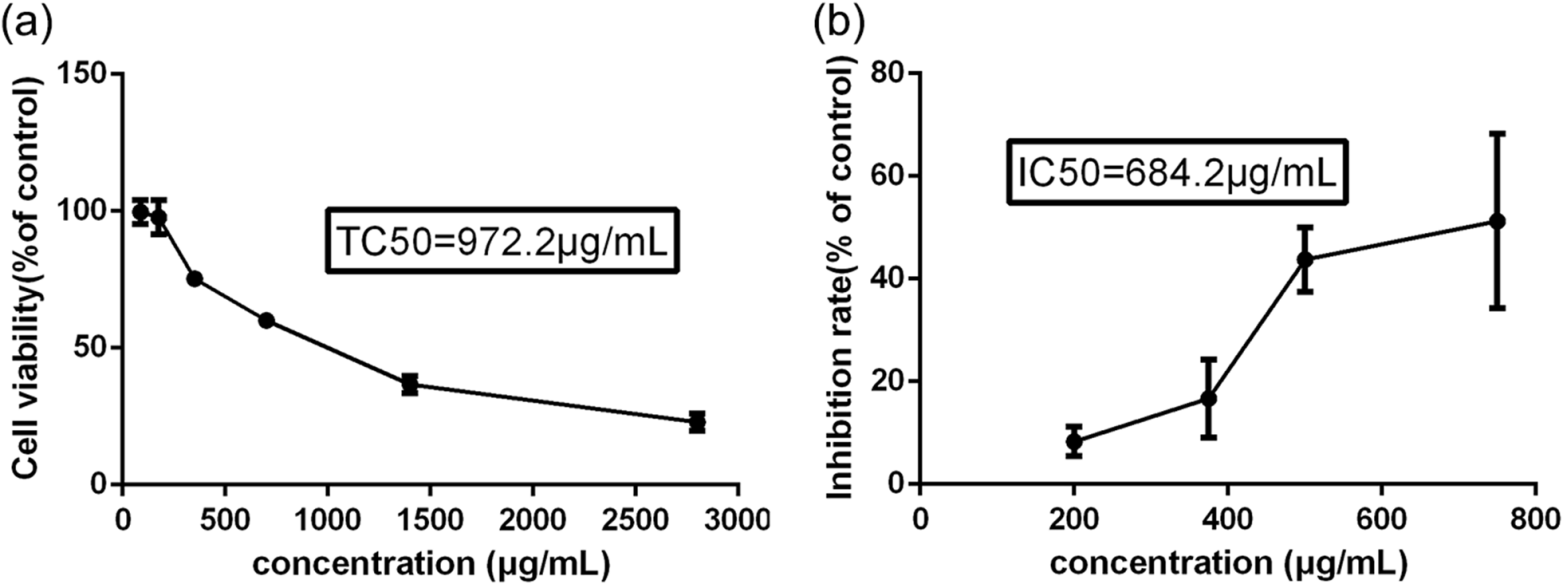
Cytotoxic and antiviral effect of LHQK against SARS-CoV-2 in Vero E6 cells. Data were presented as Mean ± SD

The cytotoxic and antiviral effect of LHQK against SARS-CoV-2 were determined at each test concentration. LHQK showed cytotoxicity for Vero E6 cells at concentration of 972.2 µg/mL (Fig. 2a), and inhibited the replication of SARS-CoV-2 virus with an IC50 value of 684.2 µg/mL (Fig. 2b).

### LHQK could alleviate the bodyweight loss, and reduce lung index, and HA titer in mice infected with HCoV-229E in vivo

**Fig. 3:**
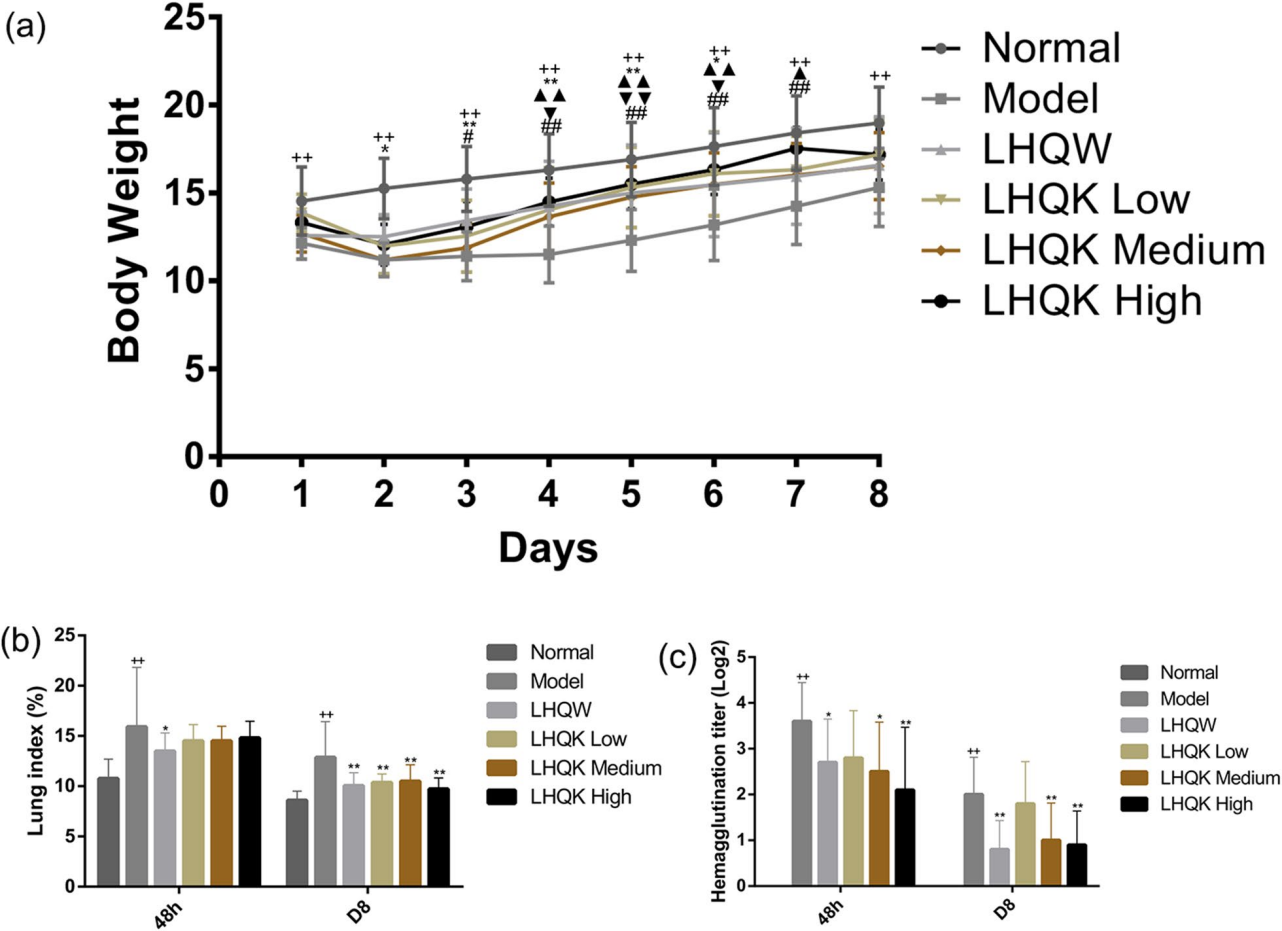
**a** Effects of LHQK on bodyweight in mice infected with HCoV-229E. Data are presented as mean ± SD (n = 10 in each group). +: model group vs normal group, ^++^P ≤ 0.01. *: LHQW group vs model group, *P ≤ 0.05, **P ≤ 0.01. ▲: LHQK Low group vs model group, ^▲^P ≤ 0.05, ^▲▲^P ≤ 0.01. ▼: LHQK Medium group vs model group, ^▼^P ≤ 0.05, ^▼▼^P ≤ 0.01. #: LHQK High group vs model as mean ± SD (n = 10 in each group). ^++^P ≤ 0.01 vs normal group. *P ≤ 0.05, **P ≤ 0.01 vs model group. **b** Effects of LHQK on lung index in animals. Date are presented as mean ± SD (n = 10 in each group). ^++^P ≤ 0.01 vs normal control. *P ≤ 0.05, **P ≤ 0.01 vs model control group. **c** The effect of LHQK on viral HA titer and the effect of LHQK on viral HA titer. Data are presented as mean ± SD (n = 10 in each group). ^++^P ≤ 0.01 vs normal group. *P ≤ 0.05, **P ≤ 0.01 vs model group

As shown in Fig. 3a, HCoV-229E infection could cause weight loss, compared with the normal group (P ≤ 0.01). Compared with the model group, the bodyweight of HCoV-229E-infected mice in the LHQW group significantly increased from day 2 after infection (P ≤ 0.05 or P ≤ 0.01), and the bodyweight of HCoV-229E-infected mice treated with LHQK at each dose significantly increased. It was suggested that oral administration of LHQK and LHQW could alleviate the weight loss of HCoV-229E-infected mice.

To evaluate the impact of LHQK on the lung index and HA titer in the mice infected with HCoV-229E, we collected the whole lung tissues of HCoV-229E-infected mice at 48 h and D8 after medication. As shown in Fig. 3b, c, compared with the normal group, the lung index and HA titer were significantly increased in the model group at 48 h and D8 (P ≤ 0.01). Compared with the model group (Fig. 3b), the lung index was significantly reduced in the LHQW group at 48 h (P ≤ 0.05), and the lung index were significantly reduced in the LHQW, LHQK Low, LHQK Medium, and LHQK High groups on D8 (P ≤ 0.01). The HA titer were significantly reduced in the LHQW, LHQK Medium, and LHQK High groups at 48 h and D8 (P ≤ 0.01 or P ≤ 0.05), compared with the model group (Fig. 3c).

### LHQK could significantly alleviate the lung tissue damage caused by HCoV-229E infection in vivo

**Fig. 4:**
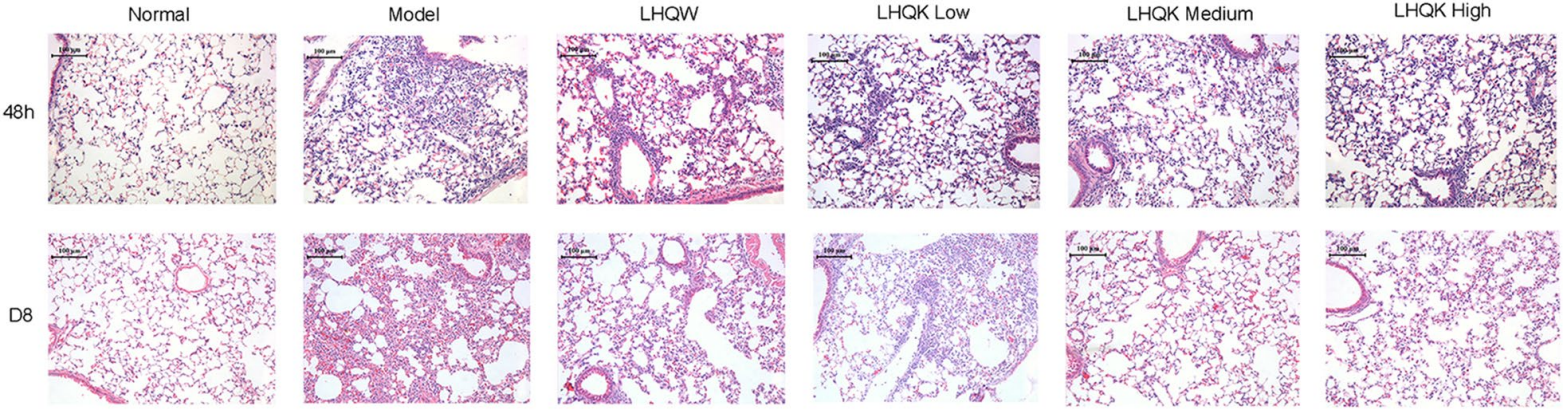
Histopathological changes in lung tissue of mice infected with HCoV-229E at 48 h and D8 after viral challenge as examined by HE staining (×100)

To evaluate the effect of LHQK on the lung tissue damage caused by HCoV-229E infection, we examined the morphological and histopathological changes among groups by H&E staining. As shown in the model group (Fig. 4), the acute lung tissue damages caused by HCoV-229E infection were present at 48 h and D8, including hemorrhage, damaged alveolar epithelium, collapse of pulmonary stent, and infiltration of inflammatory cells. All these pathological features got improved in the LHQW, LHQK Low, LHQK Medium, and LHQK High groups on D8. The degree of inflammatory infiltration and hemorrhage in the LHQW, LHQK Low, LHQK Medium, and LHQK High groups were reduced at 48 h compared with the model group. These results demonstrated that LHQK could alleviate the lung tissue damage caused by HCoV-229E infection.

### LHQK could reduce the expression of pro-inflammatory cytokines in HCoV-229E-infected mice in vivo

**Fig. 5:**
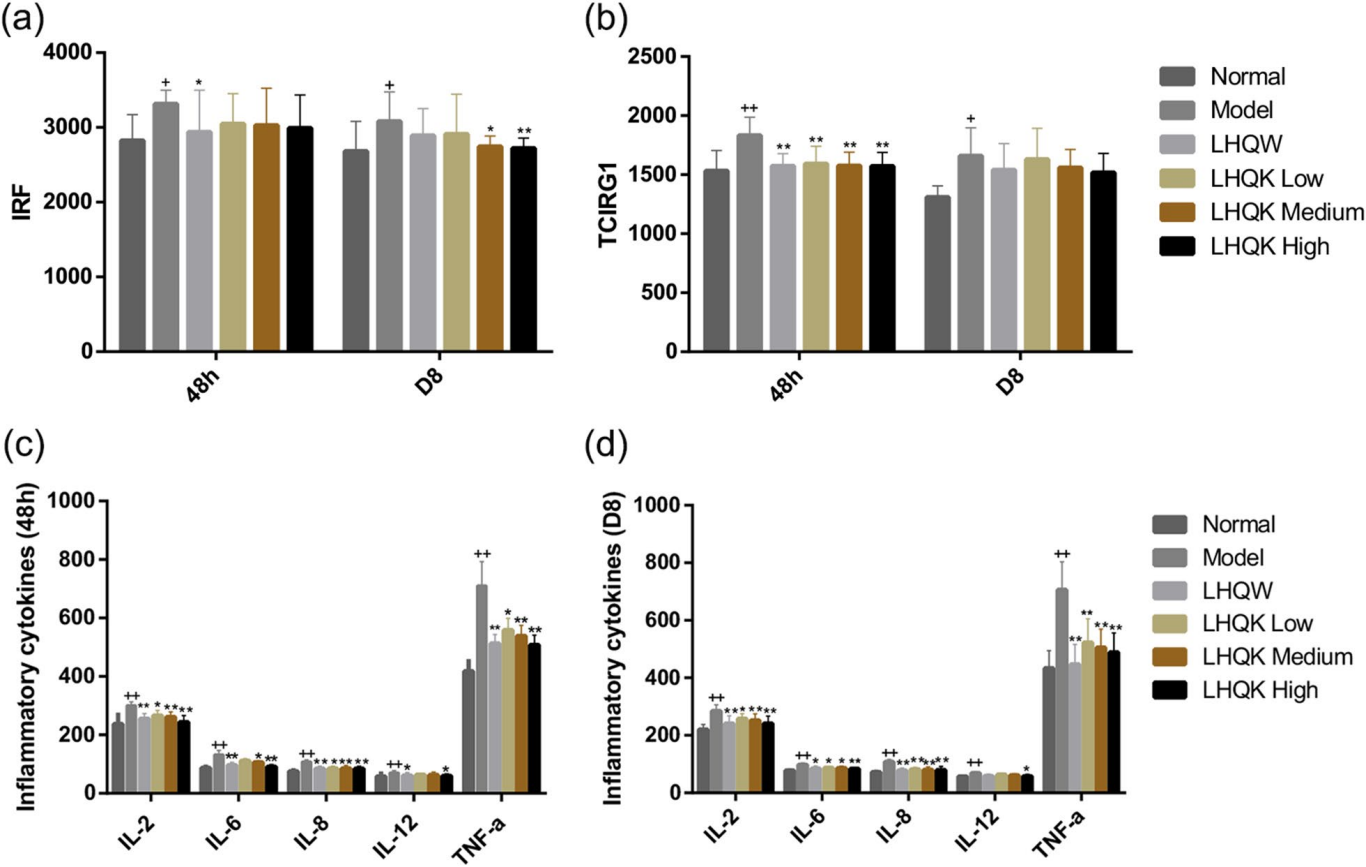
**a**, **b** Effects of LHQW and LHQK on the expression of IRF, TCIRG1 in peripheral blood of HCoV-229E-infected mice at 48 h and D8 after viral challenge. Data are presented as Mean ± SD (n = 10 in each group). ^+^P ≤ 0.05, ^++^P ≤ 0.01 vs normal group. *P ≤ 0.05, **P ≤ 0.01 vs model group. **c**, **d** Effects of LHQW and LHQK on the expression of IL-2, IL-6, IL-8, IL-12 and TNF-α in lung tissue of infected mice at 48 and D8 after viral challenge. Data are presented as Mean ± SD (n = 8 in each group). ^+^P ≤ 0.05, ^++^P ≤ 0.01 vs normal group. *P ≤ 0.05, **P ≤ 0.01 vs model group

To evaluate the expression of pro-inflammatory responses in HCoV-229E-infected mice, we used ELISA kits to determine the level of IL-2, IL-6, IL-8, IL-12, and TNF-α in the lung tissue and the expression of IRF, TCIRG1 of peripheral blood. As shown in Fig. 5a, b, IRF and TCIRG1 significantly increased in the model group as compared with the normal group (P ≤ 0.05 or P ≤ 0.01), suggesting that coronavirus (HCoV-229E) could promote the release of IRF and TCIRG1. The level of IRF was reduced in the LHQW group at 48 h (P ≤ 0.05) and reduced in the LHQK Medium and LHQK High groups on D8 compared with the model group (P ≤ 0.05 or P ≤ 0.01) (Fig. 5a). The level of TCIRG1 was significantly reduced in the LHQW, LHQK Low, LHQK Medium, and LHQK High groups at 48 h compared with the model group (P ≤ 0.01) (Fig. 5b). However, there was no difference in the TCIRG1 level among the LHQW, LHQK Low, LHQK Medium, and LHQK High and model group on D8 (Fig. 5b). As shown in Fig. 5c, d, IL-2, IL-6, IL-8, IL-12, and TNF-α increased in the model group, compared with the normal group (P ≤ 0.01). Whereas, the levels of IL-2, IL-6, IL-8, and TNF-α were decreased in the LHQW, LHQK Low, LHQK Medium and, LHQK High groups at 48 h and D8 (P ≤ 0.05 or P ≤ 0.01). IL-12 expression was reduced in the LHQK High and LHQW group at 48 h and D8 (P ≤ 0.05).

### LHQK could coordinate the Th-mediated immune responses to reduce levels of inflammation in vivo

**Fig. 6:**
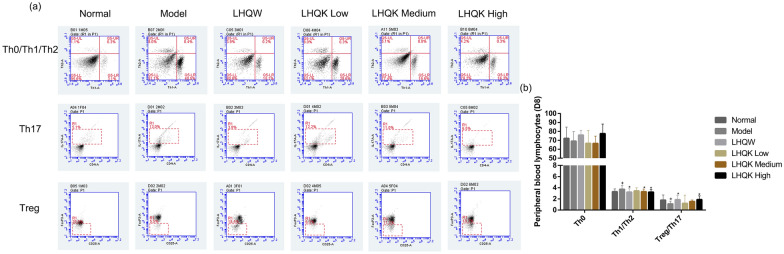
**a** The peripheral blood T helper lymphocytes population classification in different groups. **b** Effects of LHQW and LHQK on Th0, Th1/Th2 and Treg/Th17 in peripheral blood of HCoV-229E-infected mice at D8 after viral challenge. Data are presented as Mean ± SD (n = 8, in each group). ^+^P ≤ 0.05, ^++^P ≤ 0.01 vs normal group. *P ≤ 0.05, **P ≤ 0.01 vs model group

To evaluate the immunomodulatory effects of LHQK, we used flow cytometry to assay the population of Th0, Th1, Th2, Treg, and Th17 in the peripheral blood. As shown in Fig. 6, LHQK lowered the Th1/Th2 ratio and increased the Treg/Th17 ratio in a dose-dependent way, compared with the model group (P ≤ 0.05), which indicated that LHQK could coordinate the T help cell-mediated immune responses to reduce levels of inflammation. There was no significant difference in the population of Th0 in all groups.

### LHQK could inhibit MUC5AC expression and increase the number of β-IV tubulin positive staining cells in the condition-cultured RTE cells treated with TNFα in vitro

**Fig. 7:**
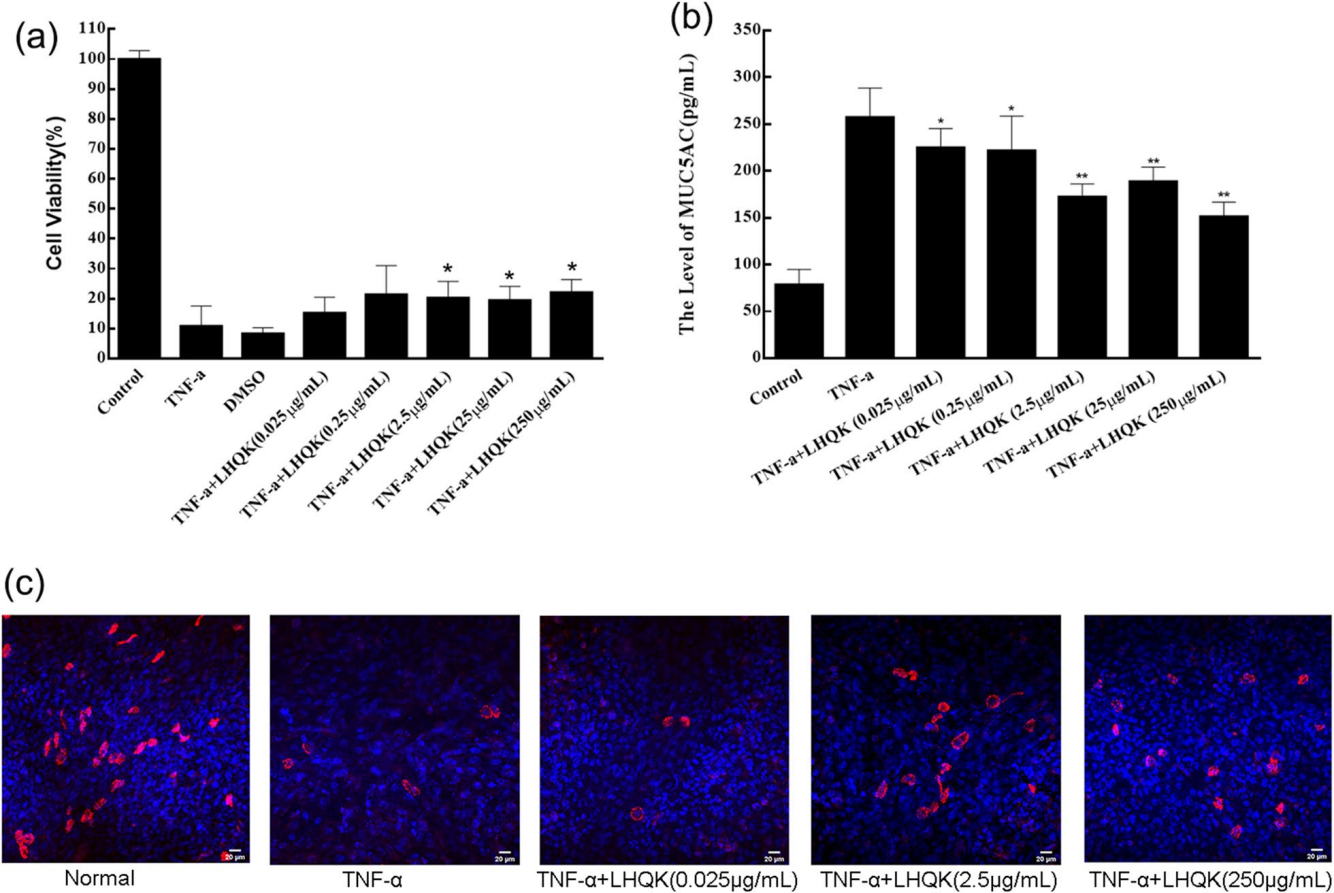
**a** Viability of 16HBE cells exposed to TNF-α with or without LHQK treatment. Data are presented as Mean ± SD (n = 3 in each group). *P ≤ 0.05 vs TNF-α group. **b** Effects of LHQK on MUC5AC expression induced by TNF-α in 16HBE cells. Data are presented as Mean ± SD (n = 5 in each group). *P ≤ 0.05, **P ≤ 0.01 vs TNF-α group. **c** Immunofluorescence labelling of airway cilia β-IV tubulin in rat bronchial primary epithelial cells (RTE). Cells were stained with anti-β-IV tubulin (ab179509, Abcam, USA) followed by Alexa 555-conjugated secondary antibody. Nuclei were counterstained with DAPI. Samples were imaged using a ZEISS LSM710. Scale bar, 20 μm

We evaluated the cell viability of 0.025, 0.25, 2.5, 25, and 250 μg/mL LHQK on 16HBE cells induced by TNF-α. The cell viability significantly decreased with the treatment of TNF-α. The cell viability increased in the TNF-α + LHQK (2.5 μg/mL), TNF-α + LHQK (25 μg/mL), and TNF-α + LHQK (250 μg/mL) groups (P ≤ 0.05) (Fig. 7a).

The expression level of MUC5AC was measure by the ELISA assay (Fig. 7b). The result showed that the level of MUC5AC was significantly increased in the cells of the TNF-α group compared with that of the control group, which was reduced by the treatment of LHQK (P ≤ 0.05 or P ≤ 0.01). Immunofluorescence staining for β-IV tubulin was used to identify the airway epithelial ciliary. As shown in Fig. 7c, positive staining cells were present in RTE cells. Compared with the control group, the positive staining of β-IV tubulin showed a decrease in the TNF-α group, which was increased by the treatment of LHQK at 2.5 μg/mL and 250 μg/mL.

### LHQK could reduce weight loss, inhibit viral replication, and alleviate lung tissue damage in the hACE2 transgenic mice infected with SARS-CoV-2 in vivo

**Fig. 8:**
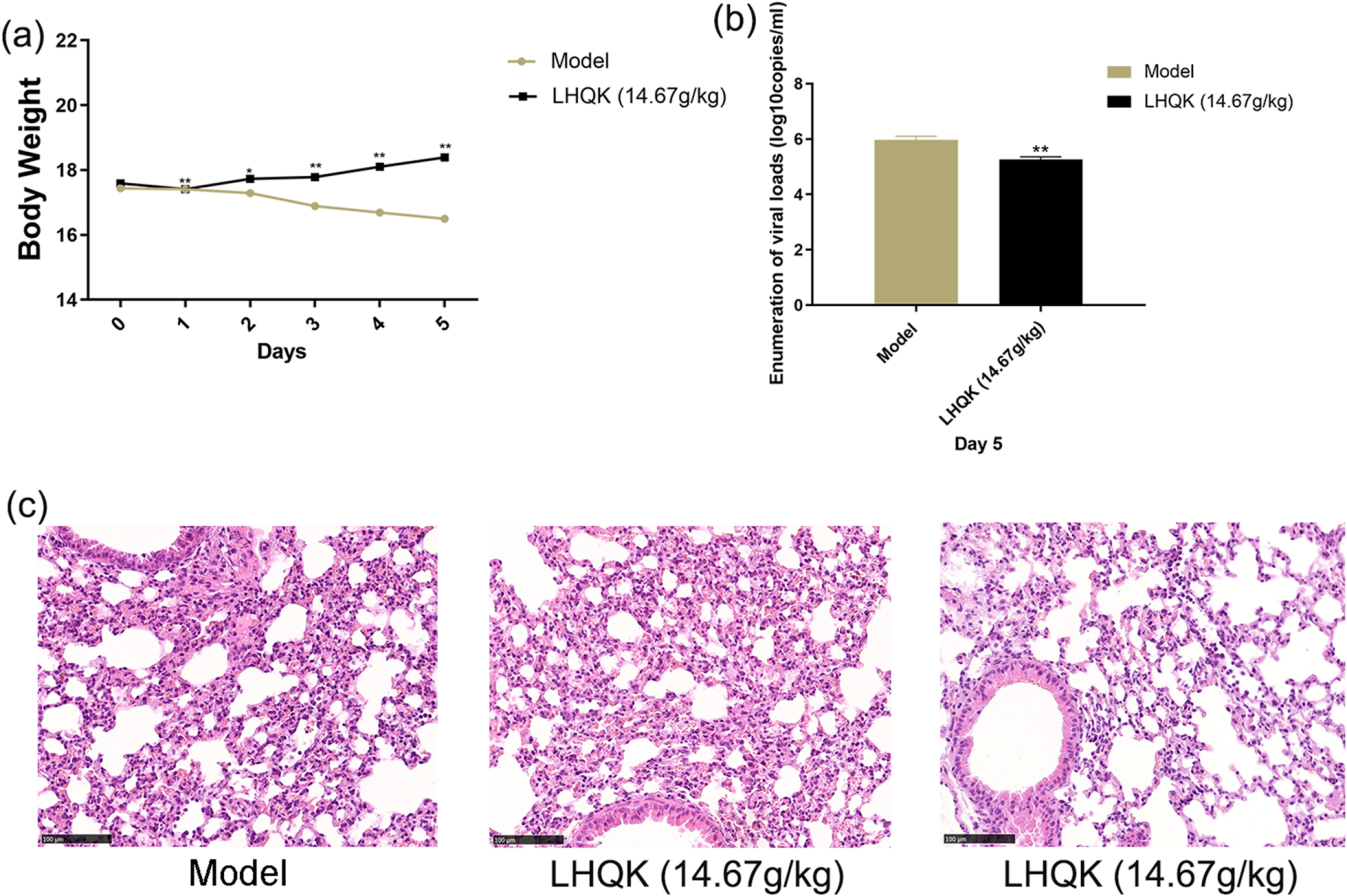
**a** Effects of LHQK on bodyweight of mice infected with SARS-CoV-2. Data are presented as Mean ± SD (n = 6 in each group). *P ≤ 0.05, **P ≤ 0.01 vs model group. **b** Effects of LHQK on viral load in lungs of SARS-CoV-2-infected hACE2 transgenic mice on day 5. Data are presented as Mean ± SD (n = 3 in each group). **P ≤ 0.01 vs model group. **c** Histopathological changes in lung tissues of SARS-CoV-2-infected hACE2 transgenic mice on day 5 after viral challenge as examined by HE staining (×200)

As shown in Fig. 8a, the bodyweight of infected mice constantly declined from day 2 in the model group. Compared with the model group, LHQK treatment reduced the bodyweight loss (P ≤ 0.05 or P ≤ 0.01), suggesting that LHQK could alleviate the clinical manifestations in the infected mice. To evaluate the antiviral effect of LHQK, the whole lung tissues of SARS-CoV-2-infected mice were collected for measurement of viral load on day 5. As shown in Fig. 8b, the viral load in the LHQK group was lower than that in the model group (P ≤ 0.01). SARS-CoV-2-infected hACE2 transgenic mice exhibited more severe damage in lung tissues than those in the LHQK group. As shown in Fig. 8c, the major histopathological features in the placebo the major histopathological features in the model group were interstitial pneumonia, which was characterized by alveolar septal thickening, leukocytes infiltration in the interstitial space, and proteinaceous debris in the alveolar space. These pathological features got slightly improved on day 5 in the LHQK group (Fig. 8c).

## Discussion

HCoVs generally causes an upper respiratory tract infection, and the clinical features of HCoVs infections are mild and self-limiting. Life-threatening pneumonia only occurs in immune-compromised individuals. However, in addition to the novel SARS-CoV-2 coronavirus, we have witnessed outbreaks of two highly pathogenic coronaviruses, SARS-CoV and MERS-CoV, over the last couple of decades. Compared with SARS and MERS, COVID-19 has spread more rapidly and emerged as a serious global public health concern [14], which reminds us that acute respiratory infections, especially caused by HCoVs, pose a continuing threat to human life.

Comparing the respiratory infections caused by HCoVs, a common pattern of virus-induced hyperinflammation can be observed in severe cases with common pathogenesis of the excessive release of proinflammatory cytokines combined with the reduction and functional exhaustion of T cells [15]. Therefore, the lessons learned from the aberrant immune responses point out that medicines with both antiviral and immunoregulatory effects could bring more clinical benefits [16]. The purpose of this study was to demonstrate whether LHQK is effective in acute bronchitis caused by HCoVs infection by inhibiting virus replication and regulating immune responses. In the mouse pneumonia model of HCoV-229E, weight loss is the most representative symptom, and the degree of reduction indicates the severity of viral infection. LHQK could prevent the progressive decline of body weight and maintain the weight gain in the HCoV-229E-infected mice, indicating that LHQK exerted therapeutic effects against HCoV-229E infection. In the lung tissues, HCoV-229E replication was paralleled by the indirect hemagglutination (IHA) antigen titers in the supernatant of lung-homogenate and reflected in the increased lung index in the Model group (Fig. 3b, c), manifested as destroyed alveolars, local atelectasis, thickened alveoli septum, and excessive infiltrating lymphocytes in the H&E staining (Fig. 4). LHQK significantly decreased the IHA antibody titer in a dose-dependent way, improved the lung histomorphology and reduced the lung index. These results revealed that LHQK had great potential for the treatment of HCoVs infection by inhibition of viral replication and inflammation. To further assess the immunoregulatory activities of LHQK, the peripheral blood T-helper cell subsets, as the key players in regulating the immune responses [17], were detected by flow cytometry, including Th1, Th2, Th17, and Treg cells (Fig. 6). The peripheral blood immunomodulatory factors and the lung tissue cytokines were evaluated by ELISA kits. The results demonstrated that LHQK lowered the Th1/Th2 ratio and increased the Treg/Th17 ratio in a dose-dependent way, which indicated that LHQK could coordinate the T help cell-mediated immune responses to reduce levels of inflammation. Moreover, the production of pro-inflammatory cytokines IL-2, IL-6, IL-8, IL-12, and TNFα and the immunoregulatory factors IRF and TCIRG1 were significantly decreased in lung tissues of LHQK groups (Fig. 5). These results indicated that Th lymphocytes that coordinated the immune response played an important role against HCoV-229E infections, by which LHQK accelerated recovery from HCoV-229E infection in the virus-infected mice.

Notably, HCoV-229E infection caused an excessive release of TNFα in mouse lung tissues, which might play a synergetic role in stimulating T cells toward Th1 polarization, and over-exuberant pro-inflammatory activities of Th1 cells cause tissue damage rather than protect the host from the virus infection, even triggering the cytokine storm [18]. To assess whether LHQK could prevent the tissue damage induced by TNFα, we treated the 16HBE cells with TNFα to establish the cell damage model and evaluated the protective effects of LHQK in vitro in the cell viability and the level of mucoprotein secretion. Compared with the model group, LHQK increased cell viability against the challenge of TNFα (Fig. 7a). To verify the increasing survival rate without the impairment of cell function, we established an air–liquid interface cultural model in RTE cells. On the one hand, the inducible capacity of motile cilia means the normal physiological function of RTE cells. On the other hand, the motile cilia on airway cells are necessary for the clearance of mucus-trapped particles out of the lung. As showed in (Fig. 7c), LHQK increased the number of positive staining cells with the antibody of β-IV tubulin in the condition-cultured RTE cells treated with TNFα. In the ELISA assay for MUC5AC expression, LHQK reduced the level of MUC5AC protein in the TNFα treated 16HBE cells, suggesting a great potential to suppress the mucus secretion of lung tissues (Fig. 7b). These results revealed that LHQK protected the epithelial cells from the damage caused by the excessive TNFα, and thus had a great potential to treat the “cytokine storm syndrome” caused by highly pathogenic coronavirus.

Compared with HCoV-229E, SARS-Cov-2 is more contagious and higher mortality. Although great hopes were placed upon COVID-19 vaccine to control the epidemic, there is still a need to identify effective treatments to avoid vaccine failure due to virus mutations. The idea of repurposing existing drugs to treat COVID-19 is an attractive strategy, TCM is one of good choices with well-established antiviral effects and safety profiles [19–21]. We re-evaluated the anti-viral effect of LHQK on the SARS-Cov-2-infected Vero E6 cells in vitro, and the result showed that LHQK had an IC_50_ value of 684.2 μg/mL (Fig. 2), and the TC_50_ of LHQK on the host cell was 972.2 μg/mL. Although, the selectivity index (SI) was greater than 1 (calculated as TC_50_/IC_50_), the window between the antiviral efficacy and the cytotoxicity indicated that the in vitro antiviral activity of LHQK was inadequate and uncertain. Given the immunoregulatory effects in in vitro and in vivo HCoV-229E infection, we hypothesized that LHQK exerted therapeutic effects on the mouse model of COVID-19. We made a pilot study to apply LHQK in the SARS-CoV-2-infected hACE mice. Compared with the model group, LHQK prevented the progressive decline of body weight, decreased the SARS-CoV-2 virus titer, and improved the lung histomorphology in the interstitial hyperplasia and inflammatory cell infiltration. The shortcoming of this study on the limited number of hACE mice was due to the limitation of bio-safety level, we could only finish a pilot study to evaluate whether LHQK was promising in the treatment of COVID-19 in vivo.

LHQK has previously shown to exhibit anti-viral effects on various respiratory viruses, including influenza, syncytial virus, etc. In this study, we found that LHQK also exerted the anti-HCoVs effects in mice infected with HCoV-229E and SARS-CoV-2. Notably, the anti-HCoV effects of LHQK might contribute to its immunomodulatory activities. Thus, we suggest that LHQK serves as a potential adjuvant combined modality therapy for HCoV infection, especially with antiviral specifics. We also consider that LHQK may be a promising therapeutic approach for emerging novel respiratory viruses.

## Conclusions

Our results demonstrate that LHQK exerts therapeutic effects on pneumonia caused by HCoVs (HCoV-229E and SARS-CoV-2) in mice, and the anti-HCoVs effects of LHQK might depend on its immunomodulatory capacities. All these results suggest that LHQK serves as a potential adjuvant for anti-HCoVs therapies.

## Data Availability

The datasets used in the current study are available from the corresponding author on reasonable request.

## Abbreviations

BSL-3: Biosafety level 3
CPE: Cytopathic effect
DMEM: Dulbecco’s Modified Eagle’s medium
FBS: Fetal bovine serum
HA: Hemagglutination
hACE2: Human angiotensin-converting enzyme 2
HCoVs: Human coronavirus
H&E: Hematoxylin & eosin
IC_50_: Median inhibition concentration
IHA: Indirect hemagglutination
LHQK: Lianhuaqingke
LHQW: Lianhua-Qingwen
MTT: Methyl thiazolyl tetrazolium
MRC-5: Human lung fibroblasts
PBS: Phosphate buffered saline
RTE: Rat bronchial primary epithelial cells
RT: Toom temperature
SARS-CoV-2: Severe acute respiratory syndrome coronavirus 2
TCID_50_: 50% Tissue culture infective dose
TCM: Traditional Chinese medicine
Vero E6: African green monkey kidney epithelial
16HBE: Human bronchial epithelial
